# The Endothelial Cell Perspective in Pulmonary Fibrosis: From Cell Fate Decisions, Intercellular Communication, and EndoMT to Emerging Therapies

**DOI:** 10.1155/carj/6653364

**Published:** 2026-06-25

**Authors:** Danni Chen, Fei Xiang

**Affiliations:** ^1^ Department of Respiratory and Critical Care Medicine, Union Hospital, Tongji Medical College, Huazhong University of Science and Technology, Wuhan, Hubei, China, hust.edu.cn

## Abstract

Pulmonary fibrosis (PF) is a progressive fibrotic interstitial lung disease characterized by aberrant activation of myofibroblasts and excessive deposition of extracellular matrix. In light of the complexity of its pathogenesis and the limited availability of approved antifibrotic drugs, PF remains a refractory lung disease imposing a substantial burden on patients. Current research into the pathogenesis of PF has largely revolved around the core hypothesis of recurrent microinjuries to alveolar epithelial cells. However, in recent years, increasing attention has been directed toward the role of endothelial dysfunction in the progression of fibrosis. This review focuses on pulmonary microvascular endothelial cells and systematically examines their regulatory mechanisms in the initiation and progression of PF from three perspectives: endothelial cell senescence and programmed cell death, intercellular crosstalk between endothelial cells and surrounding cells, and endothelial‐mesenchymal transition (EndoMT). Furthermore, we summarize antifibrotic drugs and therapeutic strategies targeting endothelial cells, providing theoretical insights and research directions to elucidate the pathogenesis of PF and explore novel therapeutic approaches.

## 1. Introduction

Pulmonary fibrosis (PF ) encompasses a group of interstitial lung diseases characterized by progressive scarring of lung tissue and declining pulmonary function. Its pathological core involves aberrant extracellular matrix deposition and excessive activation of myofibroblasts, culminating in irreversible structural damage and functional loss. The conventional model posits that repeated or persistent injury to alveolar epithelial cells serves as the initiating event in pulmonary fibrogenesis, wherein damaged epithelial cells release a range of profibrotic mediators that activate resident fibroblasts to differentiate into myofibroblasts, thereby promoting extracellular matrix accumulation. However, this epithelium‐centric paradigm may underappreciate the pivotal role of the vascular system in the fibrotic process. The lungs receive the entire cardiac output, and endothelial cells constitute approximately 30% of the lung cellular composition, forming a large dynamic barrier at the alveolar–capillary interface that not only regulates vascular permeability and gas exchange efficiency but also coordinates the maintenance of lung homeostasis through specific paracrine angiocrine factors [1]. A growing body of evidence has indicated that vascular endothelial cells are not merely passive bystanders but rather central regulatory hubs in the pathogenesis of PF [2]. Spatial transcriptomics and genetic studies have revealed early disruption at the alveolar–capillary interface in PF, including loss of endothelial integrity, which may precede fibroblast activation and macrophage recruitment [3, 4]. Single‐cell RNA sequencing further demonstrates substantial alterations in endothelial subpopulations during PF, with intensive crosstalk among epithelial cells, smooth muscle cells, fibroblasts, and macrophages, exhibiting a profibrotic signaling landscape [5]. Wu et al. identified an endothelial cell‐like myofibroblast, a transitional state that promotes the progression of PF [6]. Additional studies have confirmed the presence of vascular structural remodeling, endothelial barrier dysfunction, aberrant angiogenesis, endothelial senescence, and altered expression of endothelial‐specific markers in PF [1]. Collectively, these findings suggest that endothelial reprogramming is an active participant in fibrosis progression rather than a passive accompanying phenomenon. Therefore, a comprehensive dissection of the functional evolution of endothelial cells in PF not only deepens our understanding of disease pathogenesis but also opens new avenues for developing vascular‐targeted therapeutic strategies.

Accordingly, this review will address these questions from three key perspectives. First, we will explore endothelial senescence and programmed cell death, analyzing how these intrinsic cellular fate decisions transform endothelial cells from functional components of the barrier into persistent sources of pathological signals. Second, we will examine the societal dysfunction of endothelial cells—specifically, their aberrant communication with neighboring cells such as immune cells and fibroblasts—and elucidate how this disturbed crosstalk reshapes the immune microenvironment and activates stromal cells. Finally, we will focus on endothelial‐mesenchymal transition (EndoMT), detailing how this fundamental shift in cellular identity directly contributes to the accumulation of myofibroblasts and the aberrant deposition of extracellular matrix. These three dimensions collectively underscore the central role of endothelial cells as drivers of PF. By systematically synthesizing recent advances in these areas, this review aims to propose novel therapeutic strategies centered on restoring endothelial homeostasis, thereby charting new directions for the treatment of PF.

## 2. Endothelial Cell Senescence and Programmed Cell Death

**FIGURE 1:**
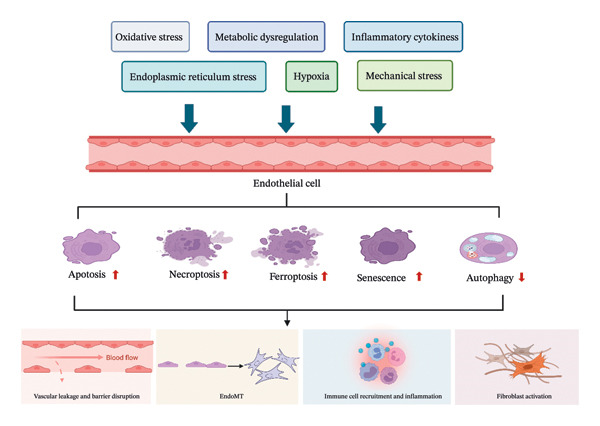
Endothelial cell senescence and programmed cell death in PF.

Endothelial cell senescence and its programmed cell death have been increasingly recognized to participate in the initiation and progression of PF. This section will focus on endothelial cell senescence, apoptosis, ferroptosis, necroptosis, and other forms of programmed cell death and review their pathological significance and related regulatory mechanisms in PF, thereby providing a basis for a deeper understanding of the role of endothelial cell dysfunction in PF (Figure 1).

Under certain conditions, endothelial cells undergo profound functional changes, including senescence, apoptosis, necroptosis, ferroptosis, and impaired autophagy. These abnormal states collectively promote PF by modulating inflammation, affecting angiogenesis and barrier function, and promoting EndoMT and fibroblast activation.

### 2.1. Endothelial Cell Senescence

Endothelial cell senescence is a critical pathological factor driving the initiation and progression of PF. It is characterized by irreversible cell cycle arrest, heightened proinflammatory responses, telomere shortening, mitochondrial dysfunction, and the release of senescence‐associated secretory phenotype (SASP) factors [7]. In the bleomycin‐induced injury model, PF in young mice gradually resolves alongside increased vascular density, whereas aged mice exhibit persistent fibrosis accompanied by capillary rarefaction and endothelial dysfunction [8]. At the molecular level, senescent endothelial cells undergo profound functional remodeling: They not only lose homeostatic functions (e.g., downregulation of eNOS/NO signaling) but also acquire an active profibrotic phenotype, including the secretion of SASP factors and an increased propensity for EndoMT [8–10]. This process is initiated by the heightened sensitivity of endothelial cells to injurious stimuli. In response to fibrogenic stimuli, endothelial cells can rapidly undergo extensive transcriptional reprogramming independently of other cell types, exhibiting characteristics of senescence and EndoMT, thereby serving as early responders to fibrosis [11]. In the context of aging, this initial response becomes further entrenched and amplified. The transcription factor ERG, a master regulator of pulmonary capillary homeostasis and repair, exhibits functional dysregulation in aged endothelial cells [12]. Loss of ERG in endothelial cells promotes paracrine activation of fibroblasts, reduces the number of normal capillary endothelial cells, and impedes the resolution of PF in young mice [12]. These findings suggest that aging‐associated chromatin remodeling in pulmonary endothelial cells leads to transcriptional dysregulation, impaired vascular repair, and sustained fibrosis following injury. Recent bioinformatic analyses have further identified four core genes closely linked to endothelial senescence and PF, including the protective factors MYCT1 and PLEKHA1, as well as the profibrotic genes PCDH12 and PLXND1 [13]. These genes directly influence the senescence status and profibrotic potential of endothelial cells, offering molecular targets for therapeutic intervention [13]. Importantly, these alterations do not occur in isolation. Through the secretion of proinflammatory and profibrotic mediators such as SASP, senescent endothelial cells engage in intricate crosstalk with adjacent senescent epithelial cells, activated fibroblasts, and immune cells, thereby establishing a complex proinflammatory and profibrotic signaling network and impeding tissue repair [10, 14].

### 2.2. Endothelial Cell Programmed Cell Death

#### 2.2.1. Endothelial Cell Apoptosis

Apoptosis is a genetically programmed form of cell death characterized by the preservation of membrane integrity and the formation of apoptotic bodies, playing an indispensable role in embryonic development, tissue homeostasis, and immune function [15]. Studies have demonstrated that in regions of severe fibrosis, pulmonary microvascular density is significantly reduced, accompanied by increased endothelial cell apoptosis [16]. Endothelial cell apoptosis not only directly contributes to microvascular loss and capillary rarefaction but also promotes fibroblast activation and extracellular matrix deposition through paracrine signaling, thereby accelerating pulmonary structural remodeling. Abnormal activation of TGF‐β signaling and elevated levels of PEDF directly or indirectly inhibit VEGF and its signaling pathways, further exacerbating endothelial cell apoptosis and leading to the loss of microvessels in fibrotic areas [16–18]. Excessive apoptosis of endothelial cells leads to the disruption of the pulmonary microvascular barrier, increased vascular permeability, and the leakage of plasma components into the pulmonary stroma, thereby exacerbating the inflammatory response and promoting the release of profibrotic factors [19]. Some apoptosis‐resistant endothelial cells may acquire a fibroblast‐like phenotype through EndoMT, directly participating in the formation of fibrotic lesions [19]. The capillary sparsification and loss of endothelial cell characteristics caused by endothelial apoptosis further exacerbate the local hypoxic microenvironment, creating a vicious cycle that continuously drives the progression of fibrosis [18]. Furthermore, several apoptosis‐targeted therapeutic strategies have demonstrated preclinical efficacy. For instance, recombinant adenosine deaminase ameliorates PF by inhibiting microvascular endothelial apoptosis and alleviating capillary rarefaction [20]. Adenoviral delivery of VEGF in a rat model of idiopathic pulmonary fibrosis (IPF) reduces endothelial apoptosis, enhances vascularization, and improves vascular remodeling [16]. However, it should be noted that VEGF itself may exacerbate PF [16]. Treatment with rhBMP9 restores the impaired BMP9/BMPR2/SMAD signaling pathway and alleviates endothelial cell apoptosis and vascular injury [21]. Ten‐eleven translocation 1 activates Kruppel‐like factor 6 expression via demethylation, subsequently upregulating the S1PR3/RhoA/ROCK axis and exacerbating lipopolysaccharide‐induced microvascular endothelial apoptosis and EndoMT, ultimately promoting PF, suggesting that ten‐eleven translocation 1 may represent a viable therapeutic target [22].

#### 2.2.2. Endothelial Cell Necroptosis

Necroptosis is a regulated form of programmed cell death mediated by key factors, including RIP1, RIP3, and phosphorylated MLKL [23]. It is characterized by plasma membrane rupture and the release of proinflammatory cytokines, morphologically resembling classical necrosis [23]. Studies have shown that necroptosis in alveolar epithelial cells and macrophages can trigger pulmonary inflammation and interstitial fibrosis through the release of damage‐associated molecular patterns (DAMPs) [23–25]. Although direct evidence of endothelial cell necroptosis in PF remains lacking, relevant findings have been reported in acute lung injury models. For instance, heat stress has been shown to induce RIP1/RIP3‐dependent necroptosis in pulmonary vascular endothelial cells via activation of the MAPK, NF‐κB, and c‐Jun signaling pathways, leading to DAMPs release and exacerbated lung injury [26]. RIPK3 has been implicated in aggravating endothelial necrosis, microvascular barrier dysfunction, inflammatory responses, and microcirculatory disturbances [27]. In a high‐dose lipopolysaccharide‐induced acute respiratory distress syndrome (ARDS) syndrome model, lung injury was primarily driven by RIPK3–MLKL‐mediated necrosis and endothelial dysfunction, a process in which the chaperone complex Hsp90/p23 plays a critical role by stabilizing the RIPK3–MLKL interaction [28]. Moreover, studies have demonstrated that endothelial‐specific knockout of MLKL attenuates liver fibrosis by disrupting the profibrotic crosstalk between endothelial cells and hepatic stellate cells via inhibition of the TGF‐β/Smad2/3 signaling pathway [29]. These findings suggest that endothelial necroptosis may contribute to PF by promoting vascular inflammation, barrier dysfunction, and activation of fibrotic signaling pathways, offering a novel perspective for future mechanistic investigations.

#### 2.2.3. Endothelial Cell Ferroptosis

Ferroptosis is an iron‐dependent form of programmed cell death characterized by glutathione (GSH) depletion, lipid peroxidation, and excessive accumulation of reactive oxygen species (ROS) [30]. In PF, elevated levels of iron and iron‐related proteins in the lung provide a pathological basis for ferroptosis [31]. Vascular endothelial cells also serve as critical targets of ferroptosis. Multiple studies have demonstrated that endothelial ferroptosis exacerbates acute lung injury [32, 33]. In terms of the underlying mechanism, the key driver of ferroptosis in endothelial cells lies in an imbalance between lipid metabolism and the antioxidant system. It has been reported that bleomycin‐treated pulmonary vascular endothelial cells exhibit ROS bursts, GSH depletion, severe vascular barrier disruption, and increased vascular leakage [34, 35]. In a murine model of bleomycin‐induced PF and in human fibrotic lung tissue, the level of dopamine‐induced phosphorylation at the Q65 site of triosephosphate isomerase 1 (TPI1) is significantly reduced, impairing TPI1’s ability to direct dihydroxyacetone phosphate toward glucose metabolism [36]. This leads to an abnormal increase in the metabolic flux toward phosphatidylglycerol synthesis and the massive accumulation of lipid peroxidation products, thereby inducing ferroptosis in endothelial cells [36]. Ferroptosis in endothelial cells leads to barrier damage and triggers an inflammatory response. Endothelial cells undergoing ferroptosis also lose their inherent proregenerative paracrine signaling, instead abnormally activating neighboring fibroblasts and inhibiting the regenerative capacity of alveolar epithelial cells, thereby shifting from regeneration to fibrosis in lung tissue [36]. Targeting endothelial ferroptosis may thus hold therapeutic potential in PF. Two sulfur‐based redox protectants, N‐acetyl‐L‐cysteine and N, N′‐bis‐2‐mercaptoethyl isophthalamide, have been shown to alleviate bleomycin‐induced endothelial oxidative stress and ameliorate endothelial dysfunction [34]. Antioxidants and iron chelators have also been reported to suppress bleomycin‐induced cytotoxicity in pulmonary vascular endothelial cells [35]. Furthermore, restoring TPI1 dopaminylation within the injured endothelial niche reverses ferroptosis, restores proregenerative angiocrine function, and alleviates PF [36].

#### 2.2.4. Endothelial Cell Autophagy

Autophagy is a conserved cellular process involving the formation of double‐membrane vesicles that engulf cytoplasmic proteins or organelles, followed by fusion with lysosomes for degradation of the encapsulated contents. Studies have shown that in a bleomycin‐induced model of PF, endothelial cells exhibit significant autophagy dysfunction, a defect considered to be a key driver of fibrosis [37]. The role of autophagy in endothelial cells is primarily protective: It maintains the endothelial phenotype and barrier function by regulating EndoMT. Specifically, the loss of the key autophagy gene ATG7 directly leads to impaired autophagic flux in pulmonary endothelial cells, causing them to lose their endothelial characteristics and acquire a mesenchymal phenotype, thereby directly participating in collagen synthesis and deposition [38]. From a signaling pathway perspective, the PI3K/AKT/mTOR axis is the central hub regulating this process [37]. Activation of this pathway inhibits autophagy, thereby promoting EndoMT, while inhibition of this pathway restores autophagy and alleviates fibrosis [37]. Furthermore, as therapeutic agents, Qimai Feiluoping Decoction and metformin have been shown to effectively inhibit EndoMT in endothelial cells by restoring autophagic activity, thereby reducing inflammatory responses and collagen deposition and consequently alleviating PF [37, 39]. Consequently, impaired endothelial autophagy is one of the key driving mechanisms of PF, and the restoration of endothelial autophagy via pharmacological intervention represents a potential therapeutic strategy for limiting and reversing the fibrotic process.

## 3. The Crosstalk Between Endothelial Cells and Neighboring Cells

**FIGURE 2:**
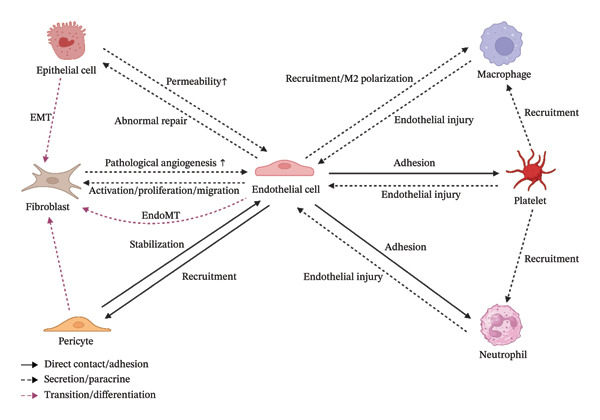
Interaction between endothelial cells and surrounding cells in PF.

The pathogenesis and progression of PF not only involve intrinsic endothelial dysfunction but also depend heavily on complex interactions between endothelial cells and various surrounding cell types. Through paracrine signaling, direct cell–cell contact, and extracellular matrix remodeling, endothelial cells interact with fibroblasts, alveolar epithelial cells, macrophages, neutrophils, pericytes, and platelets to collectively regulate inflammation, vascular remodeling, and fibrogenesis. This section will review the mechanisms underlying these interactions and their pathological significance in PF, highlighting the central regulatory role of endothelial cells in the fibrotic microenvironment (Figure 2).

Damaged pulmonary vascular endothelial cells recruit immune cells by secreting inflammatory factors, chemokines, and others. Immune cells infiltrate into the damaged area to form an immune‐dysregulated inflammatory microenvironment and induce endothelial injury. Damaged endothelial cells also bind platelets, which recruit inflammatory cells such as neutrophils and macrophages by secreting signal molecules. Alveolar epithelial cells in the pathological setting are affected by damaged endothelial cells and have difficulty initiating normal regeneration. Pericytes are also stimulated and may become one of the sources of fibroblasts or uncouple from endothelial cells, affecting normal pulmonary microvascular regenerative repair. Endothelial cells influence fibroblast activation, proliferation, and invasion through the secretion of profibrotic and proinflammatory mediators. Meanwhile, damaged endothelial cells are prone to undergoing EndoMT. Black solid lines indicate direct contact/adhesion. Black dashed lines indicate secretion/paracrine signaling. And purple dashed lines indicate transition/differentiation.

### 3.1. The Crosstalk Between Endothelial Cells and Epithelial Cells

During PF progression, a complex and dynamic crosstalk exists between pulmonary vascular endothelial cells and alveolar epithelial cells. Studies have indicated that acute lung injury, characterized by impaired epithelial barrier function, may not result directly from epithelial damage but rather from endothelial activation and dysfunction [40]. This pathological process exhibits a distinct temporal sequence. In a radiation‐induced mouse model of PF, EndoMT has been observed to occur before epithelial‐mesenchymal transition (EMT) [41]. During homeostasis and injury repair, pulmonary microvascular endothelial cells deliver a series of angiocrine factors to alveolar epithelial cells, thereby maintaining the transdifferentiation potential of type II alveolar epithelial cells as alveolar stem cells, enabling them to differentiate into type I alveolar epithelial cells upon injury and thus repair damaged alveolar structures [42]. However, during fibrogenesis, persistent aberrant signals (e.g., TGF‐β) from the damaged epithelium and the inflammatory microenvironment cause pathological reprogramming of endothelial cell phenotype and paracrine function [43, 44]. On one hand, endothelial cells directly upregulate and secrete VEGFR1 (Flt1), which through paracrine action inhibits the transdifferentiation capacity of type II alveolar epithelial cells, thereby blocking alveolar epithelial self‐repair and trapping the tissue in a vicious cycle of repetitive injury [42]. On the other hand, under repetitive injury, the expression of the protective chemokine receptor CXCR7 on pulmonary capillary endothelial cells is suppressed, leading endothelial cells to recruit VEGFR1^+^ macrophages and activate the Wnt/β‐catenin‐Jagged1 axis, indirectly exacerbating epithelial injury and aggravating PF [45]. In physiological repair, endothelial cells upregulate MMP14 via VEGFR2/FGFR1 signaling, thereby increasing the bioavailability of EGFR ligands to drive epithelial regeneration and also maintaining epithelial homeostasis through TSP1‐related mechanisms [46, 47]. These observations reflect a functional dysregulation of this intercellular communication network under the pathological condition of PF. Human tissue studies have provided direct evidence for this intercellular dialog, revealing the coexistence and interaction of endothelial‐derived myofibroblasts and epithelial‐derived basal‐like cells within the alveolar septa of early‐stage fibrotic lungs [48]. A comprehensive single‐cell sequencing study further elucidated the extensive ligand–receptor interaction network between endothelial and epithelial cells in both IPF and COVID‐19 [49]. In summary, endothelial and epithelial cells engage in dynamic and bidirectional signaling crosstalk that collectively determines whether lung tissue undergoes proper repair or progresses toward irreversible fibrosis.

### 3.2. The Crosstalk Between Endothelial Cells and Fibroblasts

Endothelial cells interact with adjacent fibroblasts not only through direct contact via intercellular junctions or membrane‐bound ligands but also by secreting profibrotic and proinflammatory mediators that regulate fibroblast activation, proliferation, and invasion [50–53]. Bian et al. discovered that FOXF1 is downregulated in endothelial cells from both IPF lungs and bleomycin‐induced PF mouse models. Endothelial‐specific loss of FOXF1 upregulates profibrotic and proinflammatory gene expression and promotes fibroblast activation, proliferation, and migration through the secretion of mediators such as IL‐6 and TNF‐α [53]. Following injury, the pulmonary vasculature in young mice initiates repair programs and facilitates fibrosis resolution [43]. In contrast, aged mice lose this reparative capacity, exhibiting transcriptional alterations that lead to the secretion of soluble profibrotic mediators, which sustain fibroblast activation [43]. Recent studies have further elucidated these mechanisms. In a silica‐induced murine model of silicosis, senescent endothelial cells highly express Galectin‐3 and release it into the microenvironment, where it directly binds to TGFBR1 on lung fibroblasts, thereby promoting their differentiation into myofibroblasts [54]. Upon exposure to volatile organic compounds, pulmonary microvascular endothelial cells secrete Gas6 following the induction of a PANoptosis‐like phenotype [55]. Gas6 subsequently binds to Axl on fibroblasts, driving fibroblast activation [55]. Atf3 has been identified as a key negative regulator of PANoptosis, attenuating volatile organic compound‐induced fibrosis [55]. In murine endothelial cells, SOX9 activates adjacent fibroblasts and promotes their migration and matrix deposition, a process partially mediated by the secreted growth factor CCN2, a direct transcriptional target of SOX9 [56]. Endothelial‐specific knockout of Sox9 reverses these fibrogenic effects [56]. Additionally, irradiated human umbilical vein endothelial cells induce fibroblast‐to‐myofibroblast differentiation via the Snail/miR‐199a‐5p axis [57]. Targeting the crosstalk between endothelial cells and myofibroblasts, Fang et al. developed a mesenchymal stem cell (MSCs)‐based therapeutic platform (MSC‐MM@LPHN) [58]. This engineered system effectively targets lung tissue, induces myofibroblast dedifferentiation, reduces the secretion of cytokines that cause endothelial injury, and prevents endothelial cells from acquiring a fibrotic phenotype, thereby restoring vascular endothelial function. Concurrently, growth factors secreted by these MSCs promote angiogenesis, ultimately leading to the reconstruction of normal vascular structures within fibrotic regions and reversal of bleomycin‐induced PF [58].

### 3.3. The Crosstalk Between Endothelial Cells and Macrophages

During the progression of PF, vascular endothelial cells become activated, transitioning from a quiescent barrier to an active hub of inflammation and immune regulation, recruiting and shaping macrophage behavior through multiple mechanisms. First, endothelial cells recruit macrophages by directly secreting chemokines and altering the local microenvironment. Suppression of the inhibitory FOXF1/R‐Ras signaling pathway in endothelial cells promotes the secretion of inflammatory mediators such as IL‐6, TNF‐α, CCL2, and CXCL1, which directly attract macrophages to sites of injury [53]. Additionally, endothelial cells upregulate MMP‐19 to activate the SDF‐1/CXCR4 axis, which not only increases vascular permeability but also facilitates macrophage transendothelial migration, thereby exacerbating fibrosis [59]. The relevance of this pathway is supported by the accumulation of CXCR4^+^ macrophages in lung tissues from patients with IPF [60]. Second, endothelial cells precisely regulate macrophage function through other modes of intercellular communication. Beyond soluble factors, endothelial‐derived CD31^+^ exosomes serve as carriers that deliver molecules such as miR‐223 to macrophages [61]. By targeting signaling proteins including RGS1, these exosomes disrupt normal immune responses, leading to macrophage subset imbalance and promoting polarization toward a profibrotic phenotype [61]. Intervention in this communication, such as supplementation with CD74^+^ exosomes, restores macrophage balance and ameliorates fibrosis, highlighting therapeutic potential [61]. Single‐cell sequencing studies have further revealed the existence of an endothelial subpopulation with high CXCL12 expression in fibrotic lungs, which forms a stable interaction network with macrophages through complex receptor–ligand pairs such as CXCL12–CXCR4, thereby sustaining a profibrotic microenvironment [62]. Senescent endothelial cells secrete galectin‐3, which activates the TLR4 receptor on macrophages and downstream NLRP3 inflammasome signaling, thereby driving fibrosis progression in a silicosis model [54].

Macrophages recruited to the site of injury are not passive participants but instead directly invade endothelial structures by releasing effector molecules and provide feedback regulation of endothelial function, thereby forming a vicious cycle with endothelial cells that drives the progression of fibrosis. A key role of macrophages is the direct disruption of endothelial barrier integrity. Activated macrophages release high levels of key inflammatory cytokines such as TNF‐α, IL‐1β, and IL‐6, which directly impair tight junctions and adherens junctions between endothelial cells, leading to markedly increased vascular permeability [63]. Macrophages can also induce more direct endothelial injury through activation of NADPH oxidase and induction of lipid peroxidation pathways, contributing to the pathogenesis of acute lung injury [64]. Macrophages actively participate in and regulate pathological vascular remodeling. Macrophages secrete multiple MMPs that degrade the extracellular matrix, releasing stored pro‐angiogenic growth factors and thereby modulating endothelial sprouting and angiogenesis [65, 66]. Among them, MMP‐12 secreted by macrophages can cleave membrane‐bound proteins on the surface of endothelial cells, such as CDH5, occludin, and TJP1, leading to endothelial cell injury and antagonizing angiogenesis [63]. The polarization state of macrophages profoundly influences the outcome of their interaction with the endothelium. Studies have demonstrated that polarized M1 macrophages enhance tube formation by irradiated endothelial cells in vitro, whereas M2 macrophages increase the adhesion of irradiated endothelial cells and promote aberrant tube formation [67]. Combined treatment with PLX3397 and nintedanib ameliorates this vascular disruption by skewing macrophage polarization toward an M1 phenotype [67]. Finally, a positive feedback loop exists between endothelial injury and macrophage recruitment. Cytokines released upon endothelial activation recruit VEGFR^+^ macrophages, which in turn secrete Wnt signals that further upregulate Jag1 expression in endothelial cells, thereby activating surrounding fibroblasts and creating a self‐amplifying fibrotic signaling cascade [45]. Conversely, inhibition of certain endothelial‐derived signals, such as von Willebrand factor deficiency, reduces macrophage migration and adhesion through suppression of the Wnt pathway and ameliorates fibrosis [68], offering a potential target for disrupting this vicious cycle.

### 3.4. The Crosstalk Between Endothelial Cells and Neutrophils

Endothelial cells serve as critical gatekeepers in initiating and orchestrating the specific recruitment of neutrophils to lung tissue. This function is primarily achieved through the expression of adhesion molecules and the secretion of chemotactic signals. Activated pulmonary microvascular endothelial cells upregulate vascular cell adhesion molecule‐1 (VCAM‐1), which mediates firm adhesion and transendothelial migration of neutrophils by binding to very late antigen‐4 (VLA‐4) expressed on their surface [69]. This mechanism has been confirmed in the bleomycin‐induced PF model, where bleomycin promotes VCAM‐1 expression on pulmonary microvascular endothelial cells, thereby enhancing neutrophil adhesion [70]. In addition to VCAM‐1, endothelial intercellular adhesion molecule‐1 (ICAM‐1) also plays a significant role in neutrophil recruitment [71, 72]. Regarding chemotactic signals, endothelial‐derived chemokines mediate neutrophil recruitment through CXCR2 receptors, a pathway regulated by G protein‐coupled receptor kinase 2 (GRK2) [73]. Nintedanib downregulates VCAM‐1 on endothelial cells, reduces CXCR2 and VLA‐4 expression on neutrophils, and upregulates GRK2, thereby effectively attenuating neutrophil chemotaxis into the lungs and ameliorating bleomycin‐induced PF [74]. Endothelial cells undergoing ferroptosis release high mobility group box 1, which promotes neutrophil extracellular traps (NETs) formation through activation of the TLR4/MYD88 pathway [75].

Suzuki et al. demonstrated that in the bleomycin‐induced PF model, pulmonary inflammation promotes neutrophil migration and NET formation in the lungs [76]. Activated neutrophils profoundly influence endothelial cell function primarily through the release of NETs and specific mediators, thereby contributing to fibrotic progression. NETs can also directly damage endothelial cells through the following multiple mechanisms. NETs induce endothelial ferroptosis via the heparan sulfate/syndecan‐1 pathway [75]. NETs activate the NLRP3 inflammasome, triggering pyroptosis in pulmonary microvascular endothelial cells [77]. Furthermore, neutrophil‐derived S100A9 serves as a key mediator of vascular remodeling, which activates the receptor for advanced glycation end products on endothelial cells and downstream PI3K/AKT signaling, leading to endothelial dysfunction [78]. NET formation depends on the activation of peptidylarginine deiminase 4 (PAD4), which catalyzes histone citrullination, driving chromatin decondensation and NET release [79]. PAD4 deficiency attenuates NET formation, prevents the reduction of vascular endothelial and epithelial cells, and inhibits the accumulation of ACTA2‐positive mesenchymal stromal cells, thereby ameliorating bleomycin‐induced PF [76].

### 3.5. The Crosstalk Between Endothelial Cells and Pericytes

During the progression of PF, endothelial cells and pericytes within the pulmonary microvasculature form a functional unit. This unit is established through direct structural coupling and complex molecular crosstalk. It plays an essential role in maintaining lung homeostasis and coordinating responses to injury [80]. Under physiological conditions, pericytes interact with endothelial cells via multiple signaling pathways. These include PDGF‐B/PDGFR‐β, angiopoietins and their Tie receptors, sphingosine‐1‐phosphate, TGF‐β, and Notch signaling. Together, they constitute a multilayered regulatory network that modulates pericyte proliferation, differentiation, positioning, and stabilization, thereby contributing to vascular architecture [80]. Structurally, pericytes establish direct contact with endothelial cells. These contacts occur in capillaries, precapillary arterioles, and postcapillary venules through shared basement membrane gaps. Tight adhesive couplings are formed via molecules such as N‐cadherin and β‐catenin, which preserve vascular wall integrity [81–84]. Functionally, endothelial cells actively recruit and anchor pericytes through continuous secretion of PDGF‐BB [85, 86]. In turn, pericytes reciprocally regulate endothelial proliferation, survival, and barrier function via paracrine signals. This bidirectional communication stabilizes microvascular structure, modulates blood flow, and maintains tissue homeostasis [87, 88].

However, within the pathological setting of PF, this cooperative relationship becomes severely dysregulated and instead propels disease progression. During the early stages of injury, endothelial cells activated by factors such as ROS undergo DNA stress and aberrant Wnt signaling within the endothelial niche, which subsequently drives pathological phenotypic changes in pericytes [89]. Activated endothelial cells release let‐7d‐deficient exosomes, which in turn activate the TGFβRI/FoxM1/Smad/β‐catenin pathway in pericytes, directly driving their differentiation into myofibroblasts and positioning them as effector cells in fibrosis [90]. Elevated VEGF in fibrotic lesions promotes abnormal endothelial sprouting and pericyte uncoupling, while increased extracellular matrix stiffness physically disrupts endothelial–pericyte interactions, leading to loss of vascular stability [91–94]. Notably, endothelial cells exert bidirectional effects on pericytes. Exosomal miR‐107 derived from endothelial cells counteracts fibrotic transformation of pericytes by inhibiting the HIF‐1α/Notch1/PDGFRβ/YAP1/Twist1 signaling axis, suggesting the presence of repair attempts at different stages of the disease [95]. Ultimately, structural decoupling and functional signaling dysregulation within the endothelial–pericyte unit collectively perpetuate a vicious cycle. This cycle involves aberrant angiogenesis, persistent inflammation, and progressive fibrotic tissue remodeling.

### 3.6. The Crosstalk Between Endothelial Cells and Platelets

In PF, the interplay between endothelial cells and platelets establishes a progressively amplifying and self‐sustaining profibrotic axis. After endothelial injury, exposed subendothelial collagen directly activates and adheres platelets. As one of the first cells to respond to injury, platelets can extravasate and release growth factors and chemokines, thereby disrupting vascular homeostasis and initiating the fibrotic cascade [96–98]. In an inflammatory environment, persistent endothelial injury and abnormal platelet activation cause platelet microparticles released from activated platelets to act back on the endothelium, inducing the expression of ICAM‐1 and vWF, promoting collagen release, and enhancing platelet adhesion to endothelial cells [99–101]. Overactivated platelets release vWF, which binds to integrin αvβ3 on the pulmonary vascular endothelial surface, activating the αvβ3‐FAK/Src signaling pathway, leading to endothelial hyperpermeability and dysfunction [102]. In even more severe cases, the activated platelet surface glycoprotein IIb/IIIa mediates firm adhesion by binding to endothelial ICAM‐1, further triggering platelet activation and releasing PDGF‐BB, thereby promoting vascular remodeling and pulmonary arterial hypertension [103].

The tripartite interaction among endothelial cells, platelets, and leukocytes forms an expanded network of inflammation and injury. Following endothelial cell injury, activated platelets bind to subendothelial collagen and express surface molecules such as P‐selectin, rapidly recruiting neutrophils, thereby forming a critical inflammatory triad of “endothelial cell‐platelet‐neutrophil” that collectively creates a profibrotic microenvironment [103]. In the context of aging, endothelial cells activate platelets via the NRP1–HIF2α–EPCR axis, prompting them to release IL‐1α, which in turn recruits profibrotic TIMP1^+^ macrophages and forms platelet–macrophage rosettes, accelerating fibrosis progression [104]. This inflammatory triad has also been corroborated in severe COVID‐19: Endothelial cell activation recruits leukocytes through the P‐selectin‐PSGL‐1 axis, promoting tissue injury and immunothrombosis [105].

## 4. EndoMT

### 4.1. Characteristics of EndoMT

EndoMT refers to the process by which endothelial cells progressively lose their specific markers, such as vascular endothelial cadherin, platelet endothelial cell adhesion molecule‐1/CD31, Tie receptors 1/2, VEGFR1/2, and coagulation factor VIII [106, 107]. Conversely, they begin to express mesenchymal cell‐specific markers, including α‐smooth muscle actin, type I and III collagen, vimentin, and fibroblast‐specific protein‐1 [106, 107]. In essence, endothelial cells can serve as a source of fibroblasts. EndoMT is also accompanied by morphological and functional changes, such as a transition from a cobblestone‐like to a spindle‐shaped morphology, reduced adhesiveness, enhanced migratory capacity, loss of polarity, and disruption of intercellular junctions [108]. In recent years, a study has identified a population of endothelial cell‐like myofibroblasts co‐expressing endothelial and mesenchymal markers in both healthy and fibrotic lung tissue and revealed a novel differentiation trajectory in normal lung tissue, whereby pulmonary endothelial cells may differentiate into EC‐like myofibroblasts that eventually develop into myofibroblasts [6]. Dysregulation of this differentiation trajectory significantly increases the production of myofibroblasts and, upon activation, promotes PF [6]. EndoMT not only provides a source of fibroblasts for fibrosis and amplifies profibrotic signals, but also causes endothelial dysfunction, microvascular barrier disruption, and vascular remodeling.

### 4.2. Triggers of EndoMT

The triggers of EndoMT include extrinsic factors such as hypoxia, oxidative stress, inflammation, and shear stress, as well as intrinsic factors, including metabolic alterations, endoplasmic reticulum (ER) stress, and autophagy. A study that explored fibroblasts in systemic sclerosis and established a hypoxia model in human lung microvascular endothelial cells confirmed the aberrant expression of nine hypoxia‐related hub genes [109]. Correlation analysis further revealed that these hypoxia‐related genes were closely associated with EndoMT markers [109]. Hypoxia has been shown to mediate EndoMT development through activation of the NF‐κB/HIF‐1α/Twist1 signaling pathway [110]. ER stress and autophagy, as intrinsic triggers, are both upregulated in the lung tissue of silicosis mice. Treatment with the autophagy inhibitor 3‐methyladenine or the ER stress inhibitor salubrinal effectively alleviates silica‐induced EndoMT [111]. Additionally, metabolic changes in endothelial cells play a significant role in EndoMT. Following treatment of human pulmonary microvascular endothelial cells with TGF‐β1 combined with IL‐1β, endothelial cells undergo EndoMT accompanied by elevated levels of carnitine palmitoyltransferase 1A and short‐chain acylcarnitines, as well as decreased levels of glycolysis/tricarboxylic acid cycle metabolites [112]. Mechanistically, fatty acid oxidation triggers EndoMT by regulating acetyl‐CoA levels, reducing inhibitory SMAD7 and enhancing SMAD2 activation [112]. Endothelial‐specific carnitine palmitoyltransferase 2 knockout mouse models further confirm that endothelial carnitine palmitoyltransferase 2 deficiency leads to EndoMT, promotes loss of endothelial intercellular junctions and barrier damage, and increases vascular permeability [112].

### 4.3. Signaling Pathways in EndoMT

Numerous signaling pathways have been implicated in EndoMT during PF, including those involving TGF‐β, IL‐1β, TNFα, Wnt, Notch, and PDGF [113, 114]. Recent studies have uncovered a range of emerging regulatory axes at various molecular levels. At the receptor and kinase signaling level, upregulation of VEGF signaling promotes the activation of the integrin–FAK pathway, thereby driving EndoMT [115]. Notably, this process can be inhibited by nintedanib [115]. APJ is a member of the G protein‐coupled receptor family, with Apelin serving as its endogenous ligand. The Apelin–APJ axis has been reported to play an important role in angiogenesis and fibrosis in multiple organs [116]. Studies have shown that in a lipopolysaccharide‐induced mouse model of PF and in cellular models derived from IPF, inhibition of the Apelin–APJ axis increases ubiquitination of angiotensin‐converting enzyme 2, thereby increasing Snail gene expression and promoting EndoMT, leading to PF [116, 117]. Meanwhile, galectin‐3 has been demonstrated to promote EndoMT by activating the PI3K/AKT pathway and its downstream AKT/GSK3β/β‐catenin axis [118, 119]. In contrast, forsythoside A inhibits this process by activating the receptor‐type tyrosine phosphatase PTPRB, which exerts negative regulatory effects [120]. At the level of cellular metabolism and energy sensing, upregulation of the endothelial NAD + metabolizing enzyme CD38 promotes EndoMT by depleting NAD+ and inhibiting SIRT3 activity, whereas specific knockout of CD38 exerts protective effects [121]. Mangiferin directly activates AMPK, enhancing the activity of the downstream FoxO3/SIRT3 signaling axis, thereby inhibiting EndoMT [122]. Furthermore, inhibition of the glucocorticoid‐metabolizing enzyme 11β‐hydroxysteroid dehydrogenase type 1 upregulates heme oxygenase‐1, subsequently suppressing TGF‐β1 or bleomycin‐induced EndoMT and ameliorating PF [123]. At the level of the extracellular microenvironment and protease systems, endothelial‐derived MMP‐19 interacts with endothelin‐1 (ET‐1) to synergistically exacerbate EndoMT progression [59]. Deficiency of vWF inhibits EndoMT by suppressing Wnt signaling, while simultaneously modulating vascular abnormalities and limiting M2 macrophage infiltration [68]. Collectively, these studies depict a regulatory landscape of EndoMT driven by the integration of multiple signaling pathways.

## 5. Drugs and Therapeutic Strategies Targeting Endothelial Cells

### 5.1. Therapeutic Drugs Targeting Endothelial Cells

#### 5.1.1. Nintedanib

Nintedanib exhibits multifaceted vascular endothelial protective effects in PF. Nintedanib ameliorates PF and pulmonary vascular remodeling by inhibiting IL‐11‐induced EndoMT, endothelial–leukocyte interactions, angiogenesis, and endothelial cell senescence through suppression of ERK1/2 phosphorylation [124]. Additionally, nintedanib inhibits bleomycin‐induced EndoMT by suppressing endothelial FAK activation [115]. Nintedanib also improves pulmonary vascular remodeling by inhibiting PDGFR and FGFR and suppresses EndoMT through downregulation of the transcription factor Twist1 [125]. Furthermore, nintedanib attenuates neutrophil chemotaxis by upregulating GRK2 and downregulating CXCR2 and VLA‐4 in neutrophils, while also reducing endothelial cell activation via decreased VCAM‐1 expression, collectively contributing to the amelioration of PF [74]. However, a recent study indicated that the antifibrotic efficacy of nintedanib is influenced by endothelial cell‐related lipids [126]. Higher levels of plasmenyl phosphatidylethanolamine 18:0p/22:6 were associated with lower 24‐month survival rates in patients with PF [126]. This suggests that PF patients with persistently elevated plasma levels of plasmenyl phosphatidylethanolamine 18:0p/22:6—characterized as the endothelial cell‐damaged type—may respond poorly to nintedanib and have a worse prognosis [126]. Although nintedanib exerts antifibrotic effects through multitarget regulation of endothelial cell function, endothelial cell damage‐related lipid metabolic features may become a limiting factor for its therapeutic efficacy, indicating that future treatment of PF should incorporate precise stratification based on endothelial cell subtypes and lipid biomarkers.

#### 5.1.2. Pirfenidone

Pirfenidone exerts its antifibrotic and vascular protective effects through multiple mechanisms. Studies have demonstrated that pirfenidone inhibits ERK1/2 phosphorylation, thereby blocking IL‐11‐induced EndoMT, endothelial–leukocyte interactions, and endothelial cell senescence, leading to improved PF and pulmonary vascular remodeling [124]. Moreover, pirfenidone attenuates early PF following lipopolysaccharide‐induced ARDS by suppressing Hedgehog signaling‐mediated EndoMT [127]. TGF‐β‐induced Rho kinase activation disrupts endothelial network formation, and pirfenidone has been shown to inhibit Rho kinase activity in TGF‐β‐stimulated cell monolayers, thereby preserving the endothelial cell network [128]. These findings suggest that pirfenidone exerts pleiotropic protective effects in inhibiting PF progression by modulating multiple signaling pathways and targeting vascular endothelial function.

#### 5.1.3. Endothelin Receptor Antagonists

ET‐1 not only exerts potent vasoconstrictive activity but also induces pulmonary vascular remodeling, recruits fibroblasts, and promotes neutrophil migration, suggesting that it plays an important role in the pathogenesis of PF [129–131]. Although endothelin receptor antagonists have been shown to protect endothelial cells and delay the progression of PF and pulmonary arterial hypertension in animal studies, they have not demonstrated significant benefits in clinical trials [132, 133]. Randomized controlled trials in patients with IPF have shown that macitentan (a dual endothelin receptor antagonist) failed to significantly improve lung function, disease progression, or time to death [134]. Similarly, bosentan did not improve lung function, quality of life, or dyspnea symptoms in IPF patients [135]. Some studies suggest that endothelin receptor antagonists may only be effective at a very early stage of fibrosis [130]. In summary, although the ET‐1/endothelin receptor axis has multiple pathogenic effects, monotherapy with endothelin receptor antagonists in patients with PF has not translated into clinical benefit, and future exploration of early intervention or combination strategies is warranted.

#### 5.1.4. Phosphodiesterase Inhibitors

Nerandomilast, a phosphodiesterase 4B inhibitor, has been shown to slow the decline in lung function in patients with IPF and progressive PF, while exhibiting a relatively favorable safety profile, leading to its approval for the treatment of both IPF and progressive PF [136, 137]. One mechanism underlying its antifibrotic effects involves reducing microvascular permeability in cytokine‐stimulated human pulmonary microvascular endothelial cells, enhancing endothelial junctions, and suppressing adhesion protein expression, thereby alleviating vascular dysfunction [138]. Single‐cell data indicate that genes upregulated by nerandomilast are enriched in various vascular endothelial cell subsets, while downregulated genes are enriched in pathological cell types such as myofibroblasts, further suggesting that its antifibrotic effects are closely associated with the restoration of endothelial homeostasis [139]. As a phosphodiesterase‐5 inhibitor, sildenafil has shown protective effects against pulmonary endothelial injury in animal models [140]. However, only a few clinical trials have suggested that it may reduce all‐cause mortality in patients with IPF [141].

### 5.2. Therapeutic Strategies Targeting Endothelial Cells

#### 5.2.1. Clearing Senescent Endothelial Cells and Inhibiting SASP

Cellular senescence is not merely a hallmark of endothelial dysfunction but an active driver that sustains the profibrotic state of endothelial cells. Multiple studies have demonstrated that senolytic therapies targeting senescent endothelial cells can partially alleviate PF [142]. These approaches fall into two main categories: Senomorphics, which target pathological SASP signaling pathways, and senolytics, which directly eliminate senescent cells that release harmful SASP factors [142]. A recent study further showed that in a bleomycin‐induced persistent PF model in aged mice, administration of circulating extracellular vesicles isolated from young mice reversed transcriptomic alterations in the aged pulmonary vasculature, promoting a more youthful endothelial transcriptional phenotype [143]. This improvement in vascular response to injury subsequently attenuated PF progression [143]. However, current antisenescence therapies lack cellular specificity. Future strategies may leverage endothelial surface markers to construct antibody‐drug conjugates for the precise delivery of senolytic agents to senescent endothelial cells.

#### 5.2.2. Blocking Aberrant Angiocrine Signaling and Maintaining Homeostasis of Endothelial Microenvironment Communication

In PF, endothelial cells engage in pathological crosstalk with surrounding cells, establishing a profibrotic microenvironment. Targeting aberrantly activated angiogenic signaling pathways in endothelial cells may represent an effective therapeutic approach. Recent studies have identified a subpopulation of highly glycolytic and proliferative endothelial cells in fibrotic lung tissue characterized by Pfkfb3^+^Rhoj^+^Pdgfb^+^ expression [144]. These cells mediate ineffective intussusceptive angiogenesis and secrete profibrotic angiocrine factors [144]. Inhibition of Rhoj signaling normalizes the angiogenesis pattern, attenuates PF, and restores lung regenerative capacity [144]. Stem cell‐based engineering approaches for vascular microenvironment restoration have also demonstrated antifibrotic potential. This strategy employs ROS‐responsive lipid polymer nanoparticles anchored on the surface of MSCs to co‐deliver metformin and macitentan, achieving favorable lung targeting [58]. The antifibrotic mechanisms include induction of myofibroblast dedifferentiation, reduction of cytokine release that damages endothelial cells at its source, inhibition of endothelial cell transition toward a fibrotic phenotype, and direct protection of endothelial homeostasis [58]. Additionally, growth factors secreted by MSCs promote physiological angiogenesis rather than pathological vascular leakage [58]. These engineered MSCs ultimately establish normal vascular structures in fibroblast‐rich regions, reversing bleomycin‐induced PF [58]. Therefore, maintaining the communication homeostasis of the endothelial microenvironment and breaking the malignant crosstalk within this microenvironment may represent a new target for antifibrotic therapy, a strategy that still requires validation through clinical trials or translational studies.

#### 5.2.3. EndoMT Reversal and Re‐Endothelialization

EndoMT is fundamentally a plastic alteration of endothelial cells under pathological microenvironments, and its inherent reversibility provides the theoretical basis for inducing re‐endothelialization in endothelial cells that have undergone mesenchymal transition [145]. In recent years, multiple studies have demonstrated the feasibility of reversing endothelial cell phenotypes through interventions targeting key signaling pathways. Endothelial‐specific CD38 knockout activates the NAD+‐SIRT3 signaling pathway, which not only inhibits TGF‐β/Smad3‐mediated EndoMT progression but also significantly reduces NOX1‐mediated oxidative stress, thereby restoring endothelial characteristics and attenuating fibrosis in bleomycin‐induced PF mouse models [121]. Qimai Feiluoping Decoction restores autophagy levels by inhibiting the PI3K/AKT/mTOR pathway, effectively reversing EndoMT and ameliorating vascular dysfunction and remodeling in both in vivo and in vitro models [37]. Furthermore, nintedanib and induced pluripotent stem cell‐derived conditioned medium suppress focal adhesion kinase activity, effectively inhibiting EndoMT and restoring expression of the endothelial marker VE‐cadherin [146]. Collectively, these findings suggest that targeting key regulatory nodes of EndoMT to induce re‐endothelialization of mesenchymal phenotypic endothelial cells represents an effective strategy for restoring endothelial homeostasis and halting fibrotic progression.

## 6. Conclusion

Endothelial cells play an indispensable and pivotal role in the pathogenesis of PF. As essential components of the blood‐gas barrier, endothelial cells are not only directly involved in maintaining gas exchange function but also deeply participate in the initiation and progression of fibrogenesis through multiple mechanisms, including senescence and programmed cell death, EndoMT, and intricate crosstalk with surrounding cells. Under pathological stimuli such as inflammation, oxidative stress, and mechanical forces, endothelial cells undergo distinct cell fate decisions, including senescence, apoptosis, necroptosis, and ferroptosis. These processes disrupt endothelial barrier function while releasing proinflammatory and profibrotic mediators. Through EndoMT, endothelial cells acquire a fibroblast‐like phenotype and directly contribute to extracellular matrix deposition and vascular remodeling. As a central hub of the vascular‐immune interface, endothelial cells mediate inflammatory activation and immune cell recruitment, forming complex paracrine signaling networks with fibroblasts, epithelial cells, and pericytes that collectively drive the establishment and maintenance of a profibrotic microenvironment.

Given the central regulatory position of endothelial cells in fibrotic progression, therapeutic strategies targeting endothelial cells and their associated signaling pathways hold considerable promise. However, the development of related therapeutics remains in early exploratory stages, with considerable progress needed before clinical translation. Future research should elucidate the spatiotemporal heterogeneity of endothelial cells within the fibrotic microenvironment and their underlying molecular regulatory networks. Particular emphasis should be placed on clarifying the upstream mechanisms driving endothelial cell behavior alterations under specific pathological conditions and the signaling pathways mediating their interactions with surrounding cells. By identifying key nodes of endothelial dysfunction, the development of precisely targeted therapeutic strategies may open new avenues for the prevention and treatment of PF.

## Author Contributions

The writing of the first draft and the preparation of materials were done by Danni Chen. Fei Xiang supervised the work throughout the process.

## Funding

The authors have nothing to report.

## Disclosure

All researchers read and approved the final manuscript.

## Conflicts of Interest

The authors declare no conflicts of interest.

## Data Availability

Data sharing is not applicable to this article as no new data were created or analyzed in this study.
